# Epidermal Growth Factor Receptor in Pancreatic Cancer

**DOI:** 10.3390/cancers3021513

**Published:** 2011-03-24

**Authors:** Melissa Oliveira-Cunha, William G. Newman, Ajith K. Siriwardena

**Affiliations:** 1 Hepatobiliary Surgery Unit, Manchester Royal Infirmary, Oxford Road, Manchester, M13 9WL, UK; E-Mail: ajith.siriwardena@cmft.nhs.uk; 2 Genetic Medicine, MAHSC, University of Manchester, St Mary's Hospital, Oxford Road, Manchester, M13 9WL, UK; E-Mail: william.newman@cmft.nhs.uk

**Keywords:** EGFR, mutation, expression, pancreatic cancer

## Abstract

Pancreatic cancer is the fourth leading cause of cancer related death. The difficulty in detecting pancreatic cancer at an early stage, aggressiveness and the lack of effective therapy all contribute to the high mortality. Epidermal growth factor receptor (EGFR) is a transmembrane glycoprotein, which is expressed in normal human tissues. It is a member of the tyrosine kinase family of growth factors receptors and is encoded by proto-oncogenes. Several studies have demonstrated that EGFR is over-expressed in pancreatic cancer. Over-expression correlates with more advanced disease, poor survival and the presence of metastases. Therefore, inhibition of the EGFR signaling pathway is an attractive therapeutic target. Although several combinations of EGFR inhibitors with chemotherapy demonstrate inhibition of tumor-induced angiogenesis, tumor cell apoptosis and regression in xenograft models, these benefits remain to be confirmed. Multimodality treatment incorporating EGFR-inhibition is emerging as a novel strategy in the treatment of pancreatic cancer.

## 1. Introduction

Carcinoma of the pancreas is an aggressive disease with a poor prognosis and an overall five-year survival rate of less than 3%, making it the fourth leading cause of cancer related mortality in the Western world [1]. Surgical therapy is presently the only therapeutic modality associated with long-term survival in pancreatic adenocarcinoma. However, only about 20% of patients with pancreatic adenocarcinoma have resectable cancers, 40% have locally advanced tumors with a further 40% having co-morbidity precluding surgery or metastatic disease [2].

The dismal prognosis and the lack of effective therapeutic regimens for pancreatic cancer are related to several factors; in particular, pancreas cancer exhibits an aggressive biological phenotype characterized by early invasion of surrounding structures and rapid metastatic spread and is relatively resistant to radiation therapy and/or chemotherapy [3]. To improve the prognosis for patients with pancreatic cancer, the diagnosis should be made at an early and potentially curable stage and more biological markers for the early detection of pancreatic cancer are needed [4].

In the last two decades, many laboratories have focused effort on characterizing the molecular alterations that are present in pancreatic cancer. Much attention has been paid to the role of growth factors and growth factor receptors in pancreatic cancer. They have been implicated in carcinogenesis by affecting a variety of functions including cell proliferation, cell invasion and metastasis, angiogenesis, immune responsiveness and extracellular matrix formation. A number of growth factors and their receptors have been shown to play an important role in pancreatic cancer, including the epidermal growth factor (EGF) family. This family of ligands and receptors plays an important role in the pathogenesis of pancreatic ductal carcinoma and contributes to its aggressiveness [5].

The epidermal growth factor receptor (EGFR) is a receptor tyrosine kinase of the ERB-B family that is abnormally activated in many epithelial tumors, including non-small cell lung cancer (NSCLC). These trans-membrane proteins are activated following binding with peptide growth factors of the EGF-family of proteins. Several mechanisms lead to the receptor's aberrant activation observed in cancer, including receptor over-expression, mutation of ligand-receptor dimerization and ligand-independent activation [6].

Given the emerging importance of EGFR expression in pancreatic cancer, this report provides an overview of current knowledge in this area with emphasis on the molecular biology of EGFR. In addition, it covers the mechanisms that lead to inappropriate activation of EGFR including: activating mutations/polymorphisms, receptor overexpression, and/or loss of their negative regulatory pathways.

## 2. Methods

A computerized search of the Medline, Embase and PubMed databases from 1970 to October 2010 was undertaken. The search terms used were “pancreas” or “pancreatic” in combination with “cancer” and “EGFR”. The search yielded 277 hits.

The PubMed search was revised to produce a population of 89 reports. The abstracts of these reports were then retrieved and studied. Studies were retained if there were reports of incidence of EGFR mutation, expression, survival rates and response to treatment Abstracts from scientific meetings have been analyzed and evidence has been included in the text.

## 3. Results and Discussion

### 3.1. Molecular Biology of EGFR

EGFR is a 170 kd glycoprotein, which is commonly expressed in normal and malignant tissues and is involved in cellular communication. The four receptors of the EGF family are composed of an extracellular domain, a hydrophobic transmembrane region and a tyrosine kinase-containing cytoplasmic region. The classical EGFR receptor is also known as HER-1 (human epidermal growth factor receptor 1) or ERBB-1 (v-erb-b2 erythroblastic leukemia viral oncogene homolog 1) [6]. The other members of EGF family are ERBB2 (also termed HER2 or HER2/neu), ERBB3 (also termed HER3), and ERBB4 (also termed HER4), they all share the same molecular structure [7,8].

**Figure 1.**
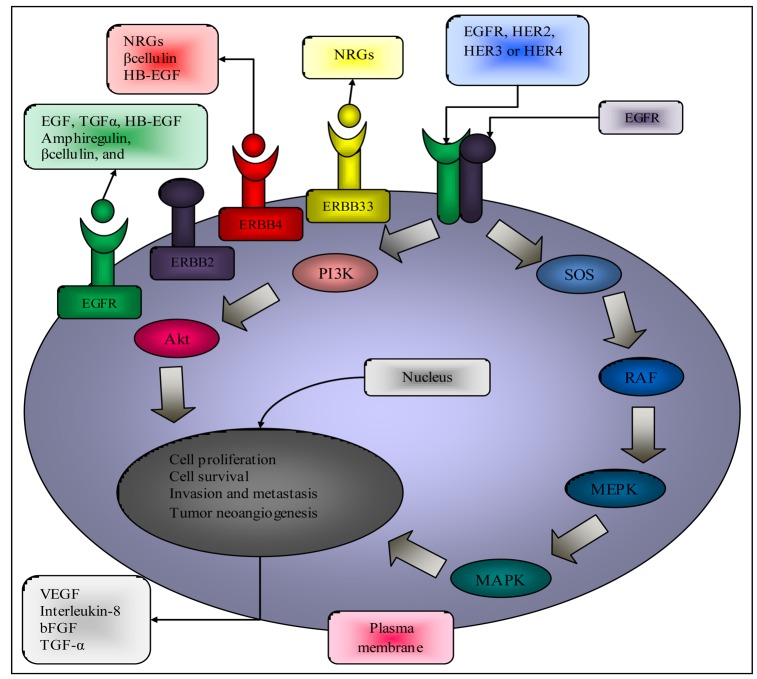
Signal Transduction Pathways controlled by the activation of epidermal growth factor receptors (EGFR). Three steps can be defined in the activation of EGFR-dependent intracellular signaling. First, the binding of a receptor specific ligand occurs in the extracellular portion of the EGFR or one of the EGFR-related receptors (ERBB2, ERBB3 or ERBB4). Second the formation of a functionally active EGFR-EGFR dimer or an EGFR-ERBB2, EGFR-ERBB3 or EGFR-ERBB4 dimer, causes the ATP-dependent phosphorylation of specific tyrosine residues in the EGFR intracellular domain. Third, this phosphorylation triggers a complex program of intracellular signals to the cytoplasm and then to the nucleus. The two major intracellular pathways activated by EGFR are the RAS-RAF-MEK-MAPK pathway, which controls gene transcription, cell-cycle progression from G1 to S phase, and cell proliferation, and the P13K-Akt pathway, which activates a cascade of anti-apoptotic and pro-survival signals. Legend: Akt: protein kinase B, HB-EGF: heparin binding epidermal growth factor, bFGF: basic fibroblast growth factor, MAPK: mitogen-activated protein kinase P, PI3K: phosphatidylinositol 3,4,5-kinase, TGF-α: transforming growth factor receptor alpha, VEGF: vascular endothelial growth factor, SOS: factor son of sevenless (Adapted from Ciardiello *et al.* [15]).

EGFR, when situated in the transmembrane position, has an extracellular domain, which provides a ligand-binding site for EGF and transforming growth factor-alpha (TGF-α). The intracellular domain of EGFR is activated upon ligand binding triggering the EGF-mediated tyrosine kinase signal transduction pathway (Figure 1) [9].

EGF and TGF-α are believed to be the most important ligands for EGFR. Ligand binding with EGFR results in receptor homo- or heterodimerization at the cell surface followed by internalization of the dimerized receptor. After dimerization, phosphorylation of the intracytoplasmic EGFR tyrosine kinase domain occurs. Phosphorylated tyrosine kinase residues serve as binding sites for the recruitment of signaling molecules, such as RAS (Rat Sarcoma Viral Oncogene). These signaling molecules have the ability to phosphorylate other “downstream” molecules [10,11]. The activation of downstream pathways promotes cellular proliferation, angiogenesis, development of metastases and reduces apoptosis [9].

Ligand binding to epidermal growth factor receptors (EGFRs) and their subsequent dimerization induces receptor auto-phosphorylation. Several tyrosine-based motifs recruit a number of signal transducers to the phosphorylated form of EGFR (such as the adaptor proteins growth-factor-receptor bound-2 (GRB2) and Src-homology-2-containing (Shc), which are responsible for the recruitment of Ras and activation of the mitogen activated protein kinase (MAPK) cascades. Another direct substrate of ERBB1 is the signal transducer and activator of transcription-5 (STAT5) [12].

The C terminus of ERBB1 contains a recognition site for the ubiquitin ligase Cbl, whereas no site is found that can directly recruit the lipid kinase phosphatidylinositol 3-kinase (PI3K). Consistent with the specificity of its docking sites, EGFR cannot directly activate the PI3K-AKT/protein kinase B (PKB) pathway, but it couples to the RAS-MAPK pathway, as well as to the RAS-PI3K-AKT/PKB pathway. EGFR signaling is negatively regulated through ubiquitylation by Cbl [12]. The ERK cascade is regulated by intrinsic positive and negative feedback (for example, ERK negatively feeds back to RAF) and extrinsic crosstalk regulation from other kinase cascades [13].

In pancreatic cancer, EGFR is overexpressed or mutant forms are able to manipulate downstream signaling. One way to manipulate the EGFR network entails setting the level of activity just below the threshold required for the mobilization of control machineries. For example, mutant forms of EGFR frequently detected in lung cancer are characterized by a basal, ligand-independent function, which is sufficient to weakly activate downstream signals but insufficient to recruit CBL to trigger receptor degradation [13,14].

MAPK pathway strongly induces the transcription and secretion of multiple ERBB ligands. This mode of autocrine positive feedback characterizes a large fraction of human tumors of epithelial origin. For example, expression of TGF-α in colorectal tumors is associated with increased risk of developing liver metastases [13,16].

Both transcription-mediated and transcription-independent mechanisms underlie negative-feedback regulation, and both are weakened in tumors. Other negative regulators of EGFR signaling are usually lost in tumors [13].

### 3.2. EGFR Mutations

The EGFR gene has been mapped to the short arm of chromosome 7 (7p). Increased expression of EGFR in human pancreatic cancer can be associated with either structural or numerical alterations of chromosome 7 [17,18]. EGFR is encoded by the c-ERBB-1 proto-oncogene. In normal pancreas, c-ERBB-1 is expressed only in the islets of Langerhans. Nevertheless, the ERBB-1 gene is over expressed in human pancreatic cell lines in up to 85% of ductal adenocarcinomas, due to an increase in gene transcription [19].

A characteristic of pancreatic cancer is that patients accumulate numerous genetic alterations by the time of clinical presentation. KRAS mutations and EGFR gene amplification probably occur early, then p16 inactivation, while the inactivation of the TP53 and SMAD4 genes appear as late changes in pancreatic carcinogenesis [4].

**Figure 2.**
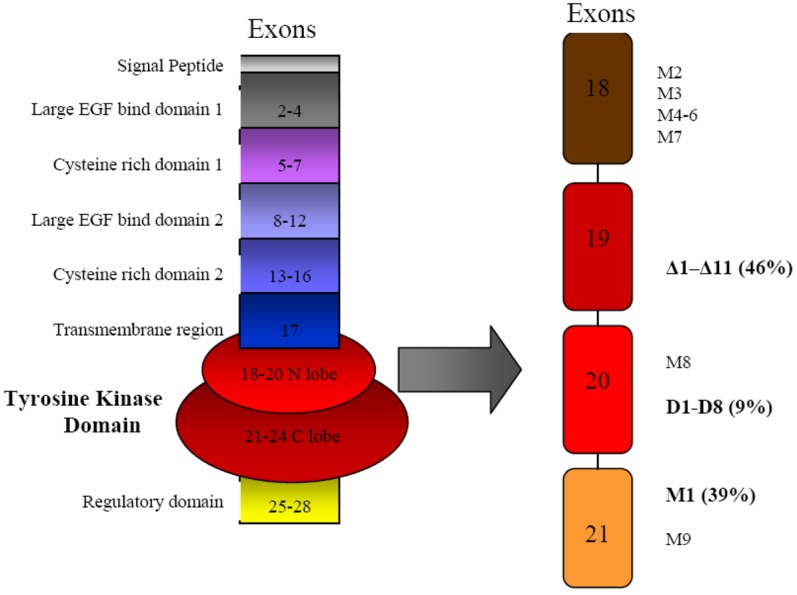
Modular structure of EGFR gene and mutations. The structure of the EGFR gene is shown on the left, and the locations and types of the mutations in the tyrosine kinase (TK) domain are shown on the right. All mutations were located within exons 18-21, which encode the N lobe and part of the C lobe of EGFR (red area of the gene on the left, which is presented in magnified form on the right). Three major types of mutations (shown in **bold**) formed 94% of the 134 mutations detected and consisted of deletions in exon 19 (labeled Δ1–Δ11), duplications and/or insertions in exon 20 (eight types labeled D1-D8), and a single-point mutation, L858R (labeled M1). The remaining 6% of mutations consisted of missense mutations in the P-loop in exon 18 (six types labeled M2-M7), exon 20 (a single type labeled M8), or exon 21 (a single type labeled M9). (Adapted from Shigematsu *et al.* [26]).

Somatic mutations in EGFR define a subset of non-small cell lung cancers, approximately 10% of cases, which are usually adenocarcinomas and bronchoalveolar carcinomas. The mutations are clustered around exons 18–21, which encode the ATP-binding pocket of the receptor, and approximately 80% consist either of a single missense mutation or nested in-frame deletions, which effect auto-homodimerization [20]. A recent study demonstrated that EGFR in-frame deletions were present in 4% (2 of 55) of patients with pancreatic cancer [20]. Highly responsive NSCLC contains somatic mutations of EGFR, including small deletions or point mutations (Figure 2). These mutations seem to result in the repositioning of crucial residues that surround the ATP-binding cleft of the EGFR tyrosine kinase domain, thereby stabilizing the interactions of the inhibitor with the kinase domain [14].

In NSLC patients EGFR mutations were not related to age or clinical stage, but there was a strong positive correlation between female gender, non-smoking status, adenocarcinoma subtype, and a high degree of differentiation to mutation presence. Across all reports, independently of ethnic origin, EGFR mutations appear almost exclusively in adenocarcinomas [21,22].

Amplification of the EGFR gene and activating mutations of the EGFR tyrosine kinase domain has been recently demonstrated to occur in carcinoma patients (NSCLC, esophageal). Interestingly, both these genetic alterations of EGFR correlated with a high probability of response to anti-EGFR agents [14,23,24].

Tzeng and collaborators analyzed EGFR mutations in pancreatic cancer cell lines and specimens from patients with pancreatic cancer who underwent resection, in 81% of the cases silent mutations were identified. This study concluded that the use of EGFR mutation status for prediction of prognosis and response to anti-EGFR therapy seems to be less useful in pancreatic cancer compared to NSCLC [25].

### 3.3. EGFR Polymorphisms

EGFR expression is regulated by one promoter region and two enhancer regions. The promoter region contains a GC-rich sequence without the characteristic TATA and CAAT boxes. The transcription starts at multiple sites within the promoter region. One enhancer element is located in direct proximity to the promoter and two others show a cooperative function: a downstream enhancer, located in intron 1 close to a polymorphic CA dinucleotide repeat, which only functions in the presence of an upstream element [27].

#### 3.3.1. EGFR intron 1 CA repeats (CA simple sequence repeat 1 – CA SSR I)

It has been demonstrated that the first intron has an important regulatory function and a highly polymorphic sequence in intron 1. A sequence repeat with 14–21 CA-repeats was first demonstrated close to the downstream enhancer by Chi and colleagues [28]. This sequence affects the efficiency of gene transcription [21].

Considering the most frequent allele containing 16 CA dinucleotide pairs as the “normal” level of EGFR transcription, modulation by an increasing number of CA pairs, up to 21, represses transcription *in vitro* by a factor of five and a decreasing number of CA pairs , down to 9, enhances transcription up to five-fold [29,30].

The *in vitro* effect translates *in vivo* to protein expression level. Allele-dependent modulation of EGFR transcription level can be observed in breast [31,32], lung [27], head and neck [33], colorectal [34], osteosarcoma and pancreas [35].

In pancreatic cancer, Tzeng and colleagues analyzed 30 microdissected pancreatic surgical specimens, matched peripheral blood samples and nine pancreatic cancer cell lines treated with erlotinib. This study concluded that shorter EGFR intron 1 CA repeat length is associated with worse pancreatic cancer clinical prognosis and *in vitro* response to erlotinib. EGFR intron 1 length can be reliably measured in peripheral blood and may translate into a quantitative predictive marker of both pancreatic cancer aggressiveness and erlotinib sensitivity [35].

In contrast, Frolov and colleagues analyzed 135 samples (50 resection samples and 85 diagnostic endoscopic ultrasound-guided fine-needle aspiration) of pancreatic adenocarcinomas. This study demonstrated that the length of the EGFR intron 1 CA repeats does not correlate with levels of EGFR expression and cannot be used as marker of clinical prognosis in pancreatic cancer patients [36].

After molecular studies in lung, breast and colorectal cancer, the assessment of the CA SSR I number of CA dinucleotide repeats and other EGFR polymorphisms as predictors of response to therapy and clinical outcome is very attractive and should be further studied in pancreatic cancer.

#### 3.3.2. Single Nucleotide Polymorphism (SNP)

SNPs are the most common sources of human genetic variation, and they may contribute to an individuals' susceptibility to cancer. Several polymorphisms have been included in databases [37]. EGFR exon 13 R521K variant has been described in other EGFR expressing tumors, such as gliomas, lung and colorectal cancer [38-40].

Studies have shown that the R497K polymorphism of the epidermal growth factor receptor (EGFR) has attenuated functions in ligand binding, tyrosine kinase activation, and growth stimulation. Wang and colleagues analyzed the effect of this polymorphism on clinicopathologic features in colorectal carcinoma patients. Their data suggest that the R497K polymorphism of the EGFR, by reducing its activation and a consequential down-regulation of its target genes, could be a key determinant for reduced tumor recurrence of advanced colorectal carcinoma patients receiving curative surgery and a longer survival of patients with advanced and metastatic colorectal carcinoma [38].

Similarly, Sasaki and colleagues analyzed EGFR mutations and/or R497K polymorphisms in NSCLC cases. In this study, EGFR mutation status was not correlated with R497K genotype of lung cancers. In node-negative patients, R497K genotype was not correlated with disease outcome. In node-positive patients, however, R497K was significantly associated with better overall survival. This association was attributable to neo-adjuvant or adjuvant chemotherapy. They have concluded that R497K polymorphism might be associated with favorable prognosis of advanced lung cancers and correlated with chemosensitivity [41].

Recent data have shown that it correlates with a decrease in EGFR phosphorylation, decreased invasion, lower nodal involvement, reduced subsequent metastasis and longer disease-free overall survival in colorectal cancer patients [38].

### 3.3. EGFR Overexpression

Recently, studies have shown EGFR overexpression in pancreatic cancer to range from 30 to 95% in various studies [42,43]. Although its expression has been correlated with local advanced and metastatic stage of disease [44], its effect on survival is controversial [42,45]. Based upon these reports and preclinical studies, novel agents directed against EGFR or its associated pathways are in development. Multiple therapeutic strategies designed to manipulate EGFR have been developed, including specific antibodies (IMC-225, Cetuximab, ABX-EGFR), flavonoid antioxidants (quercetin, luteolin), and low molecular weight EGFR-specific tyrosine kinase inhibitors (gefitinib and erlotinib) [46,47]. As trials utilizing these agents move forward, it becomes important to understand the role of EGFR expression in the pathophysiology and outcome in pancreatic cancer. Although, there is clear evidence of EGFR overexpression in pancreatic cancer, there is a lack of data on the prognostic significance of EGFR expression with reports often being contradictory [42-45,48-50].

### 3.4. EGFR and KRAS Mutations

Similar to NSCLC, pancreatic ductal adenocarcinomas exhibit a high incidence of activating KRAS mutations. The RAS protein is involved in tyrosine-kinase signal transduction pathway steps including EGFR signaling. Almost all sporadic human pancreatic carcinomata harbor a point mutation of the KRAS oncogene [51].

Studies have demonstrated that EGFR mutations and KRAS mutations were mutually exclusive in NSCLC [52,53]. Marchetti and colleagues speculated that the mutually exclusive presence of EGFR and KRAS mutations may respond to an evolutionary paradigm where EGFR activation is redundant if a mutation in KRAS is already present [52].

Immervoll and colleagues studied KRAS in relation to mutations in EGFR in pancreatic cancer. K-RAS mutations were detected in 67% of pancreatic tumors (29 out of 43 samples). No alterations in EGFR exons 18–21 were detected in KRAS-positive or KRAS-negative cases [54].

The impact of EGFR mutations, EGFR gene amplifications and KRAS mutations was studied by Lee and colleagues. Sixty six pancreatic cancer patients were included in the analysis. EGFR mutation was harbored in 1.5% of the patients. Increased EGFR copy numbers were detected in 41% of the patients. The EGFR amplifications did not significantly influence survival in pancreatic adenocarcinoma patients. KRAS mutations were identified in 49% of the cases and adversely influenced survival of pancreatic cancer patients [55].

There has been great interest in the identification of molecular markers that can predict response and survival benefit from drugs that target the epidermal growth factor receptor (EGFR). In patients with NSCLC, EGFR tyrosine kinase domain-activating mutations and EGFR gene copy number also have been reported as predictive of response and/or a survival benefit from EGFR TKI therapy [53]. More recently, mutation of the KRAS has emerged as a strong marker for lack of efficacy of antibody therapy directed against EGFR in patients with colorectal cancer, in whom the benefit appears to be limited exclusively to patients with wild-type KRAS [56].

To clarify the roles of KRAS and EGFR as predictive biomarkers in patients with advanced pancreatic cancer who received erlotinib, Da Cunha Santos and colleagues analyzed archival tumor samples for EGFR copy number and KRAS mutation status in a subset of treated and control patients who were enrolled on the double-blind, placebo-controlled study. This study concluded that in patients with advanced pancreatic cancer those who have high EGFR copy number may have shorter survival. However, the results suggest that EGFR copy number and KRAS mutation status were not identified as markers predictive of a survival benefit from the combination of erlotinib with gemcitabine for the first-line treatment of [57]. In contrast, Boeck et al have demonstrated that wild type KRAS was associated with an improved overall survival in patients with advanced pancreatic cancer [58]. The role of KRAS mutation as a predictor of resistance to EGFR TKI therapy in patients with pancreatic cancer requires more evaluation.

## 4. Conclusions

To date, several studies have explored alterations in the EGFR pathway in pancreatic cancer that are predictive factors for EGFR inhibition, such as EGFR mutations or amplifications and these reports have failed to document a meaningful prevalence of such alterations. These findings highlight the need to explore alternative explanations for aberrant EGFR pathway activation in pancreatic cancer. Mutation in the KRAS gene is unlikely to be a resistant mechanism in this disease, as opposed to lung or colorectal cancer.

Overall, EGFR is emerging as a candidate for further evaluation in pancreatic cancer. Its over-expression and the identification of activating mutations in a percentage of tumors provide a rationale for trials of anti-EGFR treatments. In turn, EGFR expression appears to play a potentially important role in modulation of tumor sensitivity to either chemotherapy or radiotherapy. Initial encouraging results of these studies need to be extended with molecular and phenotypic classification of those patients most likely to benefit.
